# Evaluation of Nine Somatic Variant Callers for Detection of Somatic Mutations in Exome and Targeted Deep Sequencing Data

**DOI:** 10.1371/journal.pone.0151664

**Published:** 2016-03-22

**Authors:** Anne Bruun Krøigård, Mads Thomassen, Anne-Vibeke Lænkholm, Torben A. Kruse, Martin Jakob Larsen

**Affiliations:** 1 Department of Clinical Genetics, Odense University Hospital, Sdr. Boulevard 29, 5000, Odense, Denmark; 2 Human Genetics, Institute of Clinical Research, University of Southern Denmark, Winsløvparken 19, 5000, Odense, Denmark; 3 Department of Pathology, Slagelse Hospital, Ingemannsvej 18, 4200, Slagelse, Denmark; Georgia Institute of Technology, UNITED STATES

## Abstract

Next generation sequencing is extensively applied to catalogue somatic mutations in cancer, in research settings and increasingly in clinical settings for molecular diagnostics, guiding therapy decisions. Somatic variant callers perform paired comparisons of sequencing data from cancer tissue and matched normal tissue in order to detect somatic mutations. The advent of many new somatic variant callers creates a need for comparison and validation of the tools, as no *de facto* standard for detection of somatic mutations exists and only limited comparisons have been reported. We have performed a comprehensive evaluation using exome sequencing and targeted deep sequencing data of paired tumor-normal samples from five breast cancer patients to evaluate the performance of nine publicly available somatic variant callers: EBCall, Mutect, Seurat, Shimmer, Indelocator, Somatic Sniper, Strelka, VarScan 2 and Virmid for the detection of single nucleotide mutations and small deletions and insertions. We report a large variation in the number of calls from the nine somatic variant callers on the same sequencing data and highly variable agreement. Sequencing depth had markedly diverse impact on individual callers, as for some callers, increased sequencing depth highly improved sensitivity. For SNV calling, we report EBCall, Mutect, Virmid and Strelka to be the most reliable somatic variant callers for both exome sequencing and targeted deep sequencing. For indel calling, EBCall is superior due to high sensitivity and robustness to changes in sequencing depths.

## Introduction

Next generation sequencing creates a wealth of genomic data and therefore efficient and accurate bioinformatic tools are required in the data analysis. Driving cancer research forward, the detection of somatic mutations in cancer samples by whole genome and exome sequencing is becoming routine in cancer research and increasingly in the clinical setting where identification of somatic mutations forms the basis for personalized medicine. Thus, highly accurate bioinformatic analysis pipelines are essential. In recent years, a number of publicly available somatic variant callers have been developed, performing paired comparisons of sequencing data from cancer tissue and matched normal tissue in order to detect somatic events. A limited number of studies have evaluated the performance of a few of these tools, many analyses are based on simulated data and evaluates only the detection of somatic single nucleotide variants (SNVs) [1–6]. Limited comparisons have been performed for the detection of somatic small deletions and insertions (indels) callers [7].

The nature of cancer tissue makes somatic variant calling a challenging task. Most solid tumors, including breast cancer are known to be admixed with a large number of stromal cells. Thus, a heterozygote allele distribution cannot be expected in cancer samples due to stromal cell admixture, cancer cell aneuploidy, large genomic amplifications and deletions and genetic heterogeneity of cancer cells due to subclonality within the cancer cell population. Hence, the sensitivity of a somatic variant caller is of great interest, in order to detect a delicate signal. An allele observed at low frequency might be just as scientifically interesting or of potential clinical importance, compared to a more frequently sequenced allele, as it may originate from a cancer subclone harboring mutations of clinical relevance. Ideally, a variant caller should also have a high specificity to keep the number of false positive calls as low as possible. The a priori odds that a given genomic position contains a somatic mutation may be as low as 1:10^5^ to 1:10^6^ as the frequency of somatic mutations in many tumors is suggested to be as low as 1–10 per Mb [8]. Hence, variant call format (VCF) files containing several thousands of somatic variant calls from exome sequencing of a tumor sample must be expected to contain a substantial amount of false positive calls, requiring additional post-call filtering. Suggestions for post-call filtering are often poorly described or not provided by the software vendors wherefore subsequent validation analysis are often necessary.

When minimizing the number of false positive calls by making strict cut-offs for parameters like Somatic Score values (SSC) (e.g. Somatic Sniper) or Fisher’s p-value (e.g. Varscan 2) one risks filtering out true low-allelic somatic events. Setting the cut-off thresholds at a reasonable point is a delicate task; balancing between not being flooded by false positives and not filtering out low-allelic true somatic calls.

Sample preparation and exome enrichment laboratory procedures can introduce PCR amplification artifacts, resulting in false positive or false negative calls. Sequencing and alignment errors, both random and systematic, also contribute to false positive calls [9,10]. For example, if a germline variant is not detected in the normal sample and successfully detected in the tumor sample the variant will be misinterpreted as a somatic variant, also resulting in a false positive call. Early attempts to identify somatic mutations relied on the “subtraction” method where independent genotyping of the tumor and normal samples were subtracted [3]. Newer algorithms utilize advanced statistical methods for the complex task of detecting somatic events. Several somatic variant callers use a Bayesian approach [8,11–15], modified in different ways, while others uses a Fisher’s exact statistics [16,17]. Each somatic variant caller is built on its own mathematical algorithm, with inherent strengths and weaknesses. The callers employ different user-defined input criteria for calling and provide varying output parameters.

Studies have shown, that sequencing errors are not uniformly distributed, as they are influenced by e.g. GC-content and platform-related phenomena such as enzyme preferences during the sequencing-by-synthesis technique [9]. The somatic variant caller EBCall [12] addresses this issue by using sequencing data from multiple non-paired normal samples as prior knowledge of the distribution of sequencing errors (and alignment artefacts) in order to optimize the discrimination between sequencing errors and genuine somatic mutations.

That current sequencing paradigms are inadequate for tumors that are impure, aneuploid or clonally heterogenous was recently demonstrated in a comprehensive study by Griffith et al [18].

The aim of our study was to compare the performance of nine different publicly available somatic variant callers, assessed to be the most commonly utilized by the bioinformatics community, for both SNV and indel calling on real-life breast cancer sequencing data. Our study evaluates the performance on medium sequencing depths by use of exome sequencing data and high sequencing depths by use of targeted deep sequencing data. Evaluating caller performances on deep sequencing data is highly relevant due to the increasing clinical use of targeted cancer panels for identification of therapeutic target mutations. We report major differences in the number of somatic calls returned by the individual callers. A large proportion of calls were returned by only a single caller and most likely represent false positive calls. Sequencing depth had clear impact on most callers, where increased sequencing depth often lead to highly improved sensitivity.

## Results

**Fig 1:**
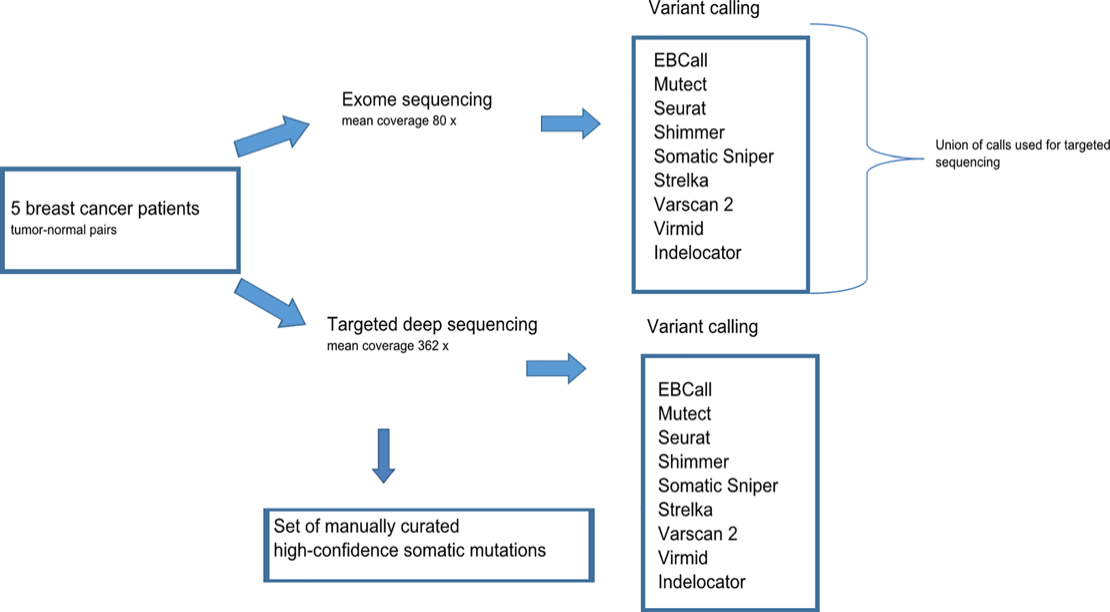
Flow chart illustrating the overall experimental design. Exome sequencing was performed on matched tumor-normal DNA from five breast cancer patients followed by somatic variant calling using nine different somatic variant callers. The union of these calls, except intronic and intergenic positions, reported by the nine somatic variant callers was included in a capture reagent and targeted deep sequencing was performed. Variant calling was repeated in the deep sequencing data. The two dataset were compared to a set of manually curated high-confidence somatic mutations which were obtained through manual inspection of the data.

In order to evaluate the performance of nine publicly available somatic variant callers the different caller software were applied to medium and high sequencing depth data, as depicted in Fig 1. We started out with exome sequencing (mean coverage 80 x) of matched tumor and normal DNA from five breast cancer patients followed by variant calling with the nine somatic variant callers. We then designed a capture reagent including the union of these calls, a total of 21,970 chromosomal regions, and performed targeted deep sequencing (mean coverage 362 x). Variant calling was repeated in the deep sequencing data. The two dataset were compared to a set of manually curated high-confidence somatic mutations which were obtained through manual inspection of the data. A detailed description is found in the methods section.

### Large variation in the number of calls returned by somatic variant callers using exome sequencing data

**Fig 2:**
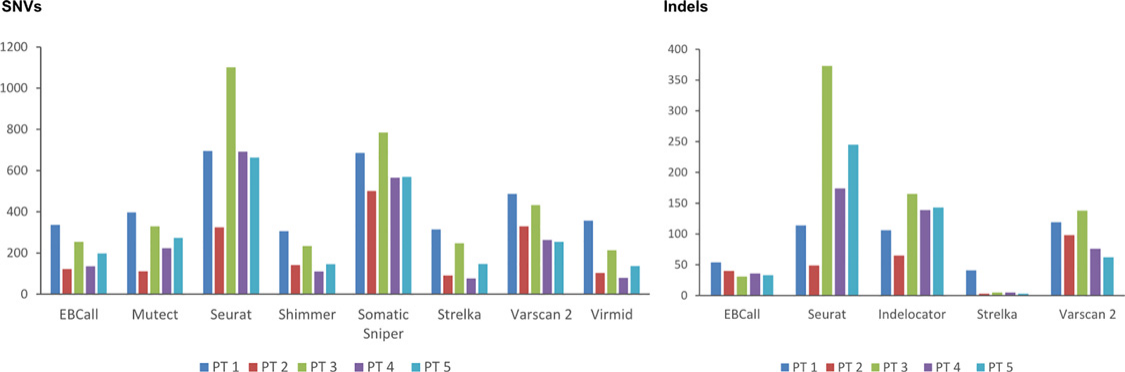
Total number of somatic mutations called by nine somatic variant caller tools in the exome sequencing data of five breast cancer samples. SNV and indel calls in left and right panels, respectively.

Four callers including Mutect, Virmid, Shimmer and Somatic Sniper return only SNVs, one caller, Indelocator, returns only indels and four callers namely Seurat, Varscan 2, EBCall and Strelka return both SNVs and indels. Somatic variant calling on the exome sequencing data of the five tumor-normal pairs revealed major differences in the number of calls returned by the different variant caller tools, as shown in Fig 2.

Seurat and Somatic Sniper returns the by far largest number of SNV calls. Strelka returns the lowest number of both SNV and indel calls. Seurat and Indelocator return surprisingly high numbers of indels. A comparable pattern is seen for the caller performances on the different tumor-normal pairs. Therefore, results from individual samples are pooled in the same dataset in the following analyses.

### Varying degree of inter-caller agreement between somatic variant callers

**Fig 3:**
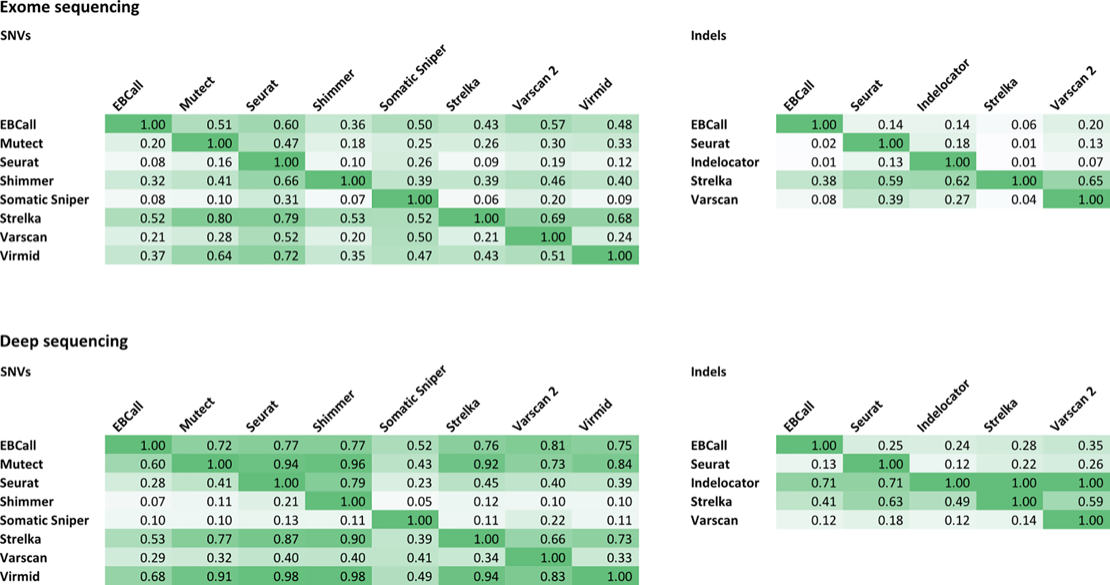
Inter-caller agreement. Pairwise comparisons of the nine studied variant callers in exome sequencing of five breast cancer samples in exome sequencing and deep sequencing data in upper and lower panels, respectively. The matrix depicts the agreement among the studied variant callers. In each horizontal line, the number reflects the fraction of calls found by the caller that are also reported by the other callers. For instance, looking at EBCall in the first line, Mutect reports 51% of the calls reported by EBCall. Deep sequencing data includes only data covered by 200 x at minimum in both tumor and normal sample. The color reflects the degree of agreement, with the highest color intensity depicting high agreement between the two callers.

The inter-caller agreement is shown in Fig 3. For SNV calling in exome sequencing data, Shimmer stands out reporting only a small fraction of the positions called by the other callers. Also displaying low agreement with the other callers, only a very small fraction of the positions called by Somatic Sniper are also reported by other callers. For indel calling in exome sequencing data, an overall low inter-caller agreement is observed. Strelka stand out from the other callers. Calls reported by Strelka are to a large extent also reported by other callers, but Strelka fails to detect a large proportion of calls detected by others, a phenomenon also seen in the calling of SNVs. Thus, Strelka is a very strict caller, which is also reflected in the low total number of calls returned by Strelka.

For both SNVs and indels, the variant callers have a higher degree of agreement in the deep sequencing data set. For SNV calling in deep sequencing data, four callers, EBCall, Mutect, Strelka, and Virmid, exhibit the highest inter-caller agreements and seem to converge towards a common set of SNV positions. A high fraction of positions reported by Shimmer are not reported by other callers. Somatic Sniper has very low agreement both in terms of detecting what is reported by other callers and for the positions reported by Somatic Sniper to be reported by other callers. For indel calling in deep sequencing data Strelka and Indelocator display the overall highest inter-caller agreement.

### Sequencing depth impacts the number of calls returned by somatic variant callers

**Fig 4:**
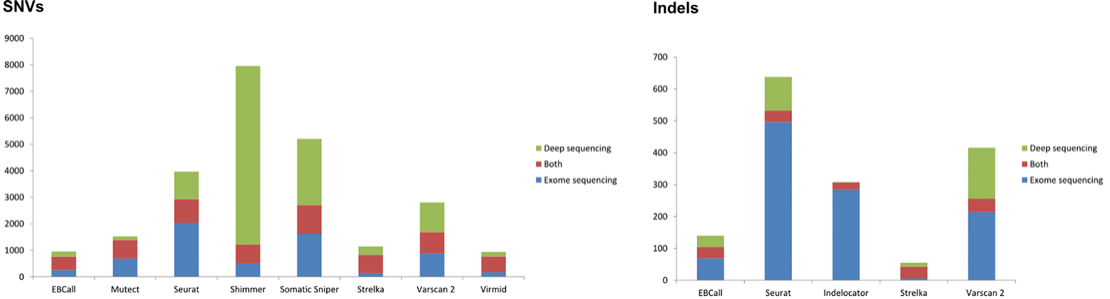
Impact on variant calling of increased sequencing depth. The impact on variant calling of increased sequencing depth for SNV and indel calling are shown in left and right panels, respectively. The number of called positions called in exome sequencing only, validation data only and both data set, depicted in blue, green and red, respectively. This analysis only includes regions that are successfully covered (at least 200 x) in the deep sequencing data.

To evaluate the effect of increased sequencing depth on the reported number of somatic calls, comparison of the number of calls returned from the exome sequencing and targeted deep sequencing data, respectively, was performed for each of the somatic callers, shown in Fig 4.

Sequencing depth was found to have markedly diverse impact on the individual callers. Shimmer and Somatic Sniper, reporting only SNVs, are highly sensitive to increased sequencing depth as these callers return a far higher number of calls from the deep sequencing data compared to exome sequencing data. These additional calls are most likely highly enriched for false positive calls as a large number are reported as recurrent mutations across the patients, shown in S1 Fig, a phenomenon not likely to be biological. Seurat and Varscan 2 also report relatively high number of calls exclusively in only one of the data set. Conversely, EBCall and Virmid and to a smaller extent Mutect and Strelka have high fractions of calls reported in both medium and high sequencing depths. Focusing on indels, Seurat, Indelocator and VarScan 2 return many positions exclusively from exome sequencing data. EBCall and especially Strelka report very few positions, but a high fraction is reported from both medium and high sequencing depths.

### Many calls are reported by only one or two callers

**Fig 5:**
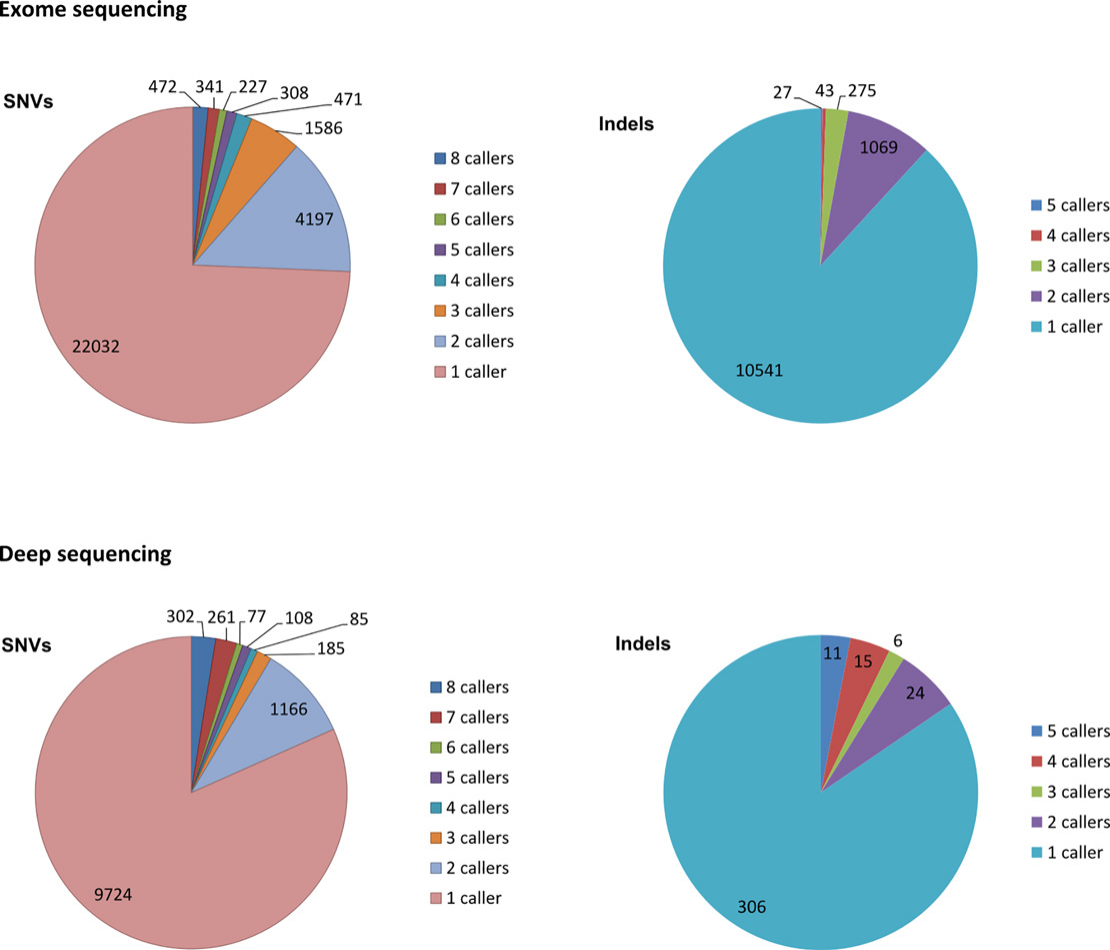
Concordance of called positions. Concordance of called positions in exome sequencing data and deep sequencing data are shown in upper and lower panels, respectively. SNVs and indels are depicted in left and right panels, respectively.

For both SNVs and indels, the main part of called positions are reported by only one or two callers as seen in Fig 5, depicting the number of callers agreeing on the called positions in exome sequencing and deep sequencing data.

In exome sequencing data, a subset comprising 472 SNV variants were detected by all eight SNV callers. In comparison, 22,032 SNVs were only called by a single caller. All five indel callers agree on 27 indels. In comparison, 10,541 indels are called by only a single indel caller. In deep sequencing data, the same tendency of many positions reported by only a single or a few callers is seen.

### The variant callers display varying degree of sensitivity

**Fig 6:**
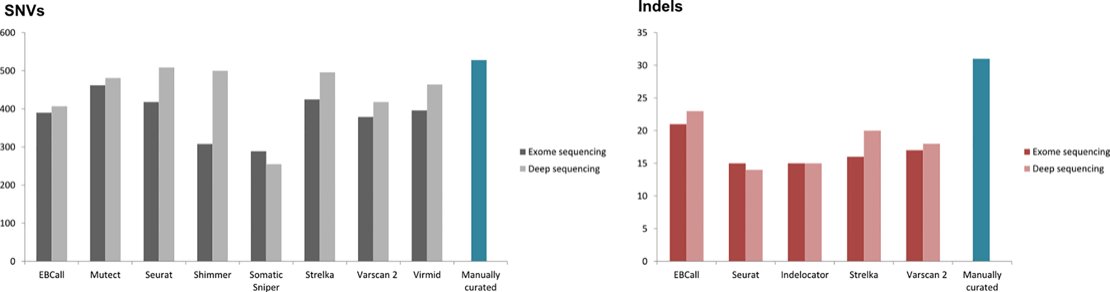
Variant caller sensitivity. Variant caller sensitivity for detecting the manually curated mutations for SNVs and indels are shown in left and right panels, respectively. The y-axis depicts the number of variant calls. The dark and light grey bars represent calls in the exome and targeted deep sequencing data, respectively.

A set of high confidence somatic variant calls within the coding region were identified as described in the methods section. The ability to detect this set of high quality variants, in total 528 SNVs and 31 indels, were used as a surrogate sensitivity measure of each of the studied somatic variant callers, depicted in Fig 6.

For SNV calling, Somatic Sniper performed poorly in both medium and high coverage data with sensitivity in exome sequencing data of 54% decreasing to 48% in the deep sequencing data. Shimmer also achieved a low sensitivity of 58% in exome sequencing data, which was highly improved in deep sequencing data to a sensitivity of 94%. Seurat, Strelka and Virmid also displayed marked improved sensitivity in deep sequencing data and Seurat attained the highest sensitivity of SNV detection of 96%. The sensitivities of EBCall and Mutect are robust to changes in sequencing depth, but only reach sensitivities of 77% and 87% in deep sequencing data, respectively. For indels, EBCall has the highest sensitivity rate of 67% and 74% in exome and deep sequencing data, respectively. The remaining four indel callers had sensitivity rates ranging between 45% and 64%.

### Major differences in calling patterns are observed among the studied somatic variant callers

**Fig 7:**
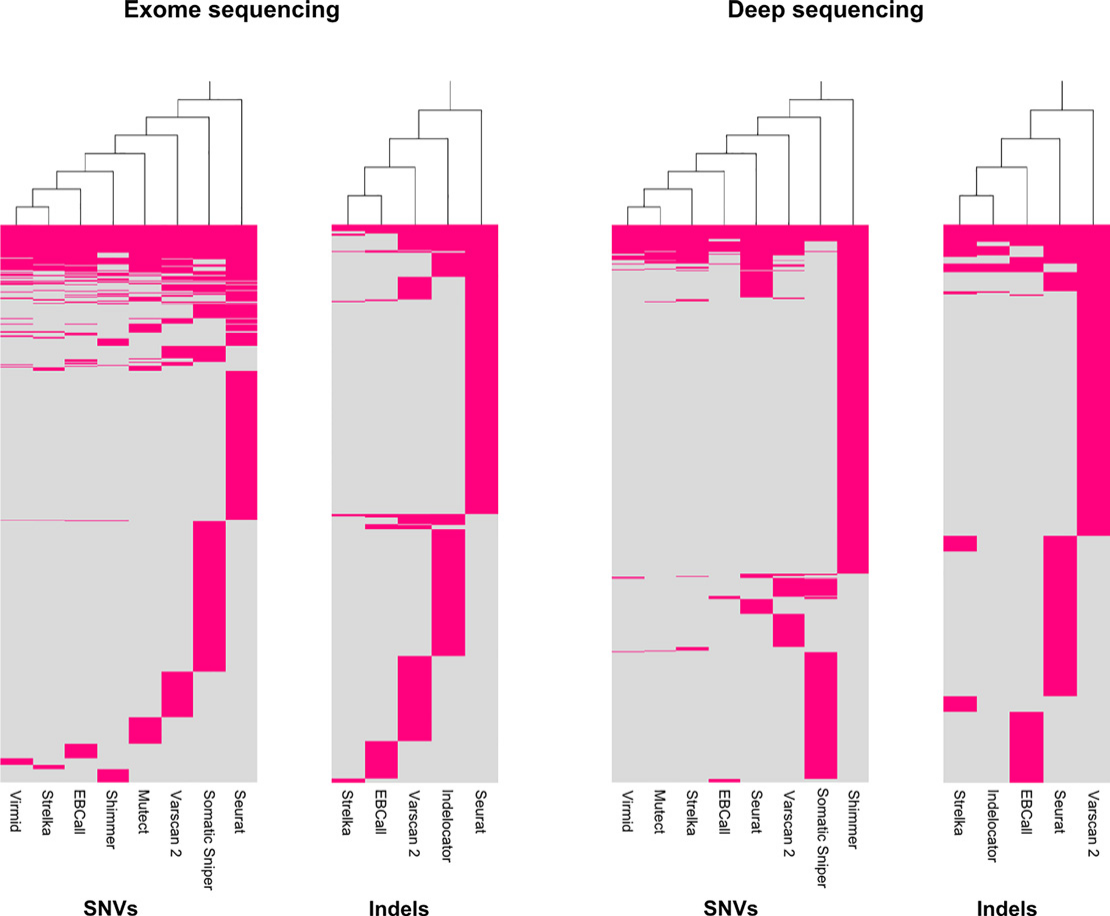
Calling patterns of the somatic variant callers. Hierarchical cluster analysis of mutations called by the somatic variant callers in exome and deep sequencing data in left and right panel, respectively. Each red line represents a called somatic mutation.

The different calling patterns of the somatic variant callers can be illustrated by hierarchical cluster analysis, as shown in Fig 7.

For SNV calling in exome sequencing data, Virmid, Strelka and EBCall display the most similar calling pattern and report relatively few positions. Conversely, Seurat and Somatic Sniper stand out with the most dissimilar calling patterns. For indel calling in exome sequencing data Strelka and EBCall have the most similar pattern, while Seurat, Indelocator and Varscan 2 report a large number of calls that are not called by other callers. Focusing on SNV calling in deep sequencing data Virmid, Mutect, Strelka and EBCall converge towards a similar pattern of mutation calling and report relatively few mutations. Shimmer and to a lesser extent Somatic Sniper, Varscan 2 and Seurat report large numbers of SNVs but in diverse calling patterns. For indel calling in deep sequencing data Seurat and Varscan 2 display a very low agreement with the other callers, reporting a number of calls exclusive to each of these callers.

## Discussion

Somatic variant callers are challenged by the balancing act between detecting true low-allelic somatic mutations and stringency of the calling procedure in order to reduce the number of false positive calls. Each caller algorithm presents its own version of this compromise and the performance is most likely dependent on the nature of the cancer samples. In contrast to most previous reports, we utilize real breast cancer sequencing datasets for evaluation of the performance of nine somatic variant callers and our data contain highly variable, low allelic fractions which is representative of breast cancer sequencing studies.

Controlling the false positive rate is the major challenge in somatic variant calling. We report the number of calls returned by the different somatic variant caller tools to be highly variable. EBCall, Mutect, Strelka and Virmid return the lowest number of SNV variant calls, display similar calling pattern in the hierarchical cluster analysis of deep sequencing data and thus converge towards the same set of mutations. Conversely, Seurat, Somatic Sniper and Shimmer (Shimmer in high sequencing depth data only) return surprisingly high numbers of SNV calls in low agreement with other callers. The very large number of positions in exome sequencing data reported by only a single or two of the nine somatic variant caller tools suggests the main part of these positions to be false positive calls, though some may represent true somatic mutations originating from a very small fraction of malignant cells. For indel calling, Seurat and Indelocator report very high numbers of returned calls, while Strelka, especially in sequencing depths around 80 x reports a very limited number of indel calls.

We observe a lower agreement in indel calling compared to SNV calling, suggesting that indel calling poses greater challenges to the callers. Correct indel calling is highly dependent on accurate alignment, and in recent updates of germline variant calling software like GATK [19] local *de novo assembly* is performed around indels in order to optimise indel calling. This method is not applied by any of the somatic variant callers included in our study.

Overall, an increase in sequencing depth results in a higher level of agreement among the different callers. For some somatic variant callers, sequencing depth highly affects the performance. For SNVs, Shimmer, Seurat and Somatic Sniper are revealed to be highly sensitive to changes in coverage and for these callers the number of returned calls is tremendously dependent on sequencing depth. Shimmer and Somatic Sniper return surprisingly high number of calls in the deep sequencing data. These positions are to a large degree not detected by any other caller and are considered to include many false positive calls. Conversely, EBCall and Virmid have high fractions of calls reported in both medium and high sequencing depths, suggesting that these algorithms are reliable in both sequencing depths around 80 x and deep sequencing data with coverages around 300 x and can thus be categorized as performing in a more robust, coverage-independent manner. Mutect performs almost similarly but has a higher false positive rate as a slightly higher fraction of calls in exome sequencing data cannot be validated in the deep sequencing data. Strelka is a conservative variant caller with a very low number of returned calls, especially at medium sequencing depths.

Focusing on indels, Seurat, Indelocator and VarScan 2, are found to be highly affected by changes in sequencing depth. Strelka returns very few calls and has the highest percentage of calls reported in both exome and deep sequencing data. EBCall is also relatively coverage-independent and performs best in “sensitivity test” detecting the manually curated positions. Seurat, Varscan 2 and Indelocator (Indelocator in medium sequencing depth only) have very high total numbers of reported calls, indicating a low specificity for these callers.

Somatic sniper has no lower cut-off for the read depth of its candidate somatic event sites and therefore the default use of Somatic Sniper returns high rates of somatic calls compared to other variant callers. Even with addition of post-call filtering of SSC > 40, a phred based score which in principle should be translated into a false discovery rate of 1 in 10,000, the resulting number of variants called by Somatic Sniper is unrealistically high. Somatic Sniper has the second-highest number of total somatic calls but nevertheless the poorest agreement with other callers and the lowest sensitivity towards the manually curated calls. Thus, Somatic Sniper most likely reports a high number of false positive calls and is, in our study, outperformed by other somatic variant callers.

In exome sequencing data, EBCall, Mutect, Strelka and Virmid detect a high fraction of the manually curated somatic mutations without also reporting a large number of suspected false positive calls. All somatic variant callers, except Somatic Sniper, detect the main part of the manually curated high-quality somatic mutations within the coding region in deep sequencing data. However, no caller detects all of them. Thus, a combination of somatic variant callers may be a good solution. A statistical approach for building a combined caller has been presented [1]. A combination of somatic variant caller tools can improve sensitivity. An open-source software tool, CAKE, that integrates four somatic variant caller algorithms, Bambino, CaVEMan, SAMtools and Varscan 2, is in fact already available [20].

Although the use of real biological data has some advantages, a major caveat to our study is the difficulty of defining the true number of somatic mutations in the cancer samples, rendering calculations of true false positive and false negative rates impossible.

Alignment of the sequenced reads greatly influences the subsequent variant calling. Thus, a possible weakness of our study is that we have used only one aligner tool, NovoAlign. The use of other alignment tools could potentially influence our results. However, NovoAlign has repeatedly been shown to be one of the best performing alignment tools available, being highly accurate. Likewise, the evaluated somatic variant callers may perform differently in other types of cancer tissue, e.g. with higher content of malignant cells. In sequencing studies of tumor samples with a higher content of malignant cells than presented here, true somatic mutations are more easily detected and thus, for high purity samples it might be beneficial to increase stringency levels and perform strict post-call filtering in order to reduce the false positive rate. It must be mentioned that it is possible that comprehensive output parameter optimization could potentially improve caller performance for some of the included callers.

## Conclusions

Our use of non-simulated sequence data has the advantage of capturing real biological variation such as the complex inherent features of cancer tissue and process errors, and therefore reveals the most realistic performance measure of the somatic mutation callers. Our study illustrates the effect of validation with targeted deep sequencing relating to the question of necessity of validation of findings from exome sequencing studies. In studies based on exome sequencing only, typically with a relatively low coverage, we recommend EBCall and Virmid as they return very few calls that cannot be validated by targeted deep sequencing.

In summary, our data reveals major differences among the nine studied somatic variant callers. EBCall, Mutect, Strelka and Virmid all perform well in our study. These four callers are seen to converge towards a common set of positions, while other of the studied variant callers report more divergent sets of positions. Sequencing depth had markedly diverse impact on individual callers. For SNV calling, we report EBCall, Mutect, Virmid and Strelka to be the most reliable somatic variant callers for both medium and high coverage sequencing data. Strelka is, however, a very conservative somatic variant caller. For indel calling, EBCall is superior compared to other callers as this caller has a high sensitivity rate, low number of returned calls and is robust to changes in sequencing depths.

## Methods

### Patient material

The study is based on fresh frozen primary tumor tissue (in one case pre-invasive tissue (DCIS)) from five breast cancer patients with invasive ductal carcinoma and matched normal tissue. Tumor tissues were secured during primary surgery and stored at -80˚C until sample preparation. Haematoxylin-eosin sections of tumor tissue were reviewed by a certified pathologist, ensuring a content of malignant cells of 60% at minimum. A start amount of 20–30 mg fresh frozen tissue was used for the purification process. Tissue disruption and homogenization was performed using TissueLyser (Qiagen) and purification of DNA was performed using AllPrep DNA/RNA Mini Kit (Qiagen). Matched normal tissue was stored as formalin-fixed paraffin-embedded (FFPE) tissue. The FFPE blocks were cut in 30–40 sections of 10 μm and DNA extracted using AS1000 Maxwell 16 (Promega, USA).

### Ethics

The study was approved by the Ethical Committee of Region Syddanmark and notified to the Danish Data Protection Agency. The patients have provided written, informed consent to participate in the study. Due to restrictions from the Ethical Committee of Region Syddanmark and in order to ensure patient confidentiality raw exome sequencing data are not available. All relevant data are available upon request to the corresponding author.

### Library construction and exome sequencing

One microgram of genomic DNA from each sample was randomly fragmented by focused acoustic shearing (Covaris inc.) according to Illumina’s protocol. The fragment length was measured by Bioanalyzer (Agilent Technologies 2100), confirming a fragment length of 150–300 bp. Exome enrichment was performed with Illumina's TruSeq DNA Sample Preparation, followed by sequencing on the Illumina HiSeq 1500 platform with paired end sequencing 2 x 100 bases, loading three exomes per lane. FASTQ files were aligned to the human reference genome GRCh37 (feb.2009) using the Novoalign v. 3 algorithm (www.novocraft.com) at default parameters. Removal of duplicate reads, recalibration and local realignment around indels were performed using Best Practices pipeline v. 2.7 [19]. The result was a mean coverage rate in the exome region of 80 x (S1 Table).

### Variant calling in exome sequencing data

On the exome sequencing data, somatic variant calling was performed using nine publicly available somatic variant callers: EBCall [12], Mutect [8], Seurat [14], Shimmer [16], Indelocator (http://www.broadinstitute.org/cancer/cga/indelocator), Somatic Sniper [13], Strelka [11], VarScan 2 [17] and Virmid [15] with default settings and recommended parameters. We did not include LOH calls in the study. Post-call filtering was applied for Indelocator, Somatic Sniper and VarScan 2. Settings and post-call filters are shown in Table 1.

**Table 1:** The nine somatic variant callers, settings and post-call filtering used in the study.

Somatic variant caller	Statistical approach	URL	V	Type of call	Settings	Post-call filter
**EBCall** [12]	Bayesian algorithm	http://gihub.com/friend1ws/EBCall	2	SNV's, INDELS	Default	-
**Mutect** [8]	Bayesian algorithm	http://www.broadinstitute.org/cancer/cga/mutect	1.1.5	SNV's	Validation_strictness Strict	-
**Seurat** [14]	Bayesian algorithm	http://sites.google.com/site/seuratsomatic	2.5	SNV's, INDELS	Phred scaled somatic score: Q > 15	-
**Shimmer** [16]	Fisher's exact test, multiple testing correction	http://www.github.com/nhansen/shimmer	0.36	SNV's	Base quality > 20, mapping quality > 10	-
**Indelocator**	Not specified	http://www.broadinstitute.org/cancer/cga/indelocator	2.3.9	INDELS	Tumor INDEL fraction > 10%	Indel seen by min 2 reads
**Somatic Sniper** [13]	Bayesian algorithm	http://gmt.genome.wustl.edu/somatic-sniper/current	1.0.3	SNV's	Mapping Quality > 10	RD: Tumor min 6, normal min 8, SSC > 40
**Strelka** [11]	Bayesian algorithm	ftp://strelka@ftp.illumina.com/	1.0.12	SNV's, INDELS	Default	-
**VarScan 2** [17]	Heuristic, Fisher's exact test	http://varscan.sourceforge.net	2.3.6	SNV's, INDELS	Strand bias [0]	Fisher's p-value < 0.05
**Virmid** [15]	Bayesian algorithm	http://sourceforge.net/projects/virmid	1.1.0	SNV's	Mapping quality > 10	-

RD: read depth. SNV: single nucleotide variant. SSC: Somatic score. V: version.

For variant calling with EBCall, exome sequencing data from unrelated individuals, run in our laboratory in a clinical setting, processed under the exact same conditions were used as prior knowledge for the purpose of discrimination between sequencing errors and somatic mutations.

A detailed description of the somatic variant callers used in the study can be found in S1 File.

### Validation of candidate somatic variants by targeted deep sequencing

The union of putative somatic mutations, except intronic and intergenic positions, reported by the nine somatic variant callers was used to select chromosomal candidate regions for targeted deep sequencing.

Additional genomic positions were included as related cancer samples from the same patients formed the basis for selecting targeted regions, described in Krøigård et al. [21], therefore the variant callers had a chance to report low allele frequency variants found in other steps of cancer progression when presented with increasing coverage.

In total, 21,970 chromosomal regions were targeted. Target enrichment was performed using SureSelect DNA enrichment methodology (Agilent). A custom SureSelect enrichment kit was designed using the Agilent SureDesign application (https://earray.chem.agilent.com/suredesign/). Library construction and SureSelect enrichment were performed according to manufacturer’s protocol and sequenced on the Illumina HiSeq 1500 platform with paired end sequencing 2 x 100 bases. Deep sequencing resulted in a mean coverage of 362 x of the targeted positions (S1 Table). Alignment and data preprocessing were performed as described above. Variant calling of the targeted sequencing data was performed by the nine variant callers using the same settings as for exome sequencing data (Table 1).

For deep sequencing data only positions covered by at least 200 x are included in the data analyses.

### Establishment of a set of high-confidence somatic mutations by an alternative method combined with manual curation

In a previous study of ours [21], an alternative calling method combined with manual curation, was applied to obtain high-confidence somatic variants from the coding regions of the included samples. To evaluate the sensitivity of the tested variant caller tools we used this conservative method to identify a set of high-confidence somatic mutations as a list of true somatic mutations. In brief, the VarScan 2 multisample tool (version 2.3.6) was utilized in its most lenient stringency settings on the validation data of the five tumor-normal pairs to generate a list of putative variants, germline as well as somatic. Subsequently, manual filtration of the variants were conducted using the following criteria: normal sample B Allele Frequency (BAF) less than 0.02, all samples should have a read depth of min. 50 x and BAF in the tumor sample should be 0.05 at minimum. Subsequently, all identified somatic mutations were manually examined by visual inspection of the BAM files to remove false positive calls as visual evaluation of called somatic mutations is increasingly recognized to be a powerful strategy for excluding false positive calls. The manual inspection was carried out as follows: BAM files of tumor and normal samples as well as five other unrelated normal BAM files were loaded in to GenomeBrowse (Golden Helix) and the postulated somatic variant calls were visualized. Variant calls in which the variant base were also present repeatedly in normal BAM files were rejected as false positive calls. As were variant calls located in repetitive areas and variants with many adjacent variants (SNP cluster regions) as they were suspected to result from systematic misalignment.

The resulting set of manually validated high-confidence somatic variants allowed us to evaluate the sensitivity, but not the specificity of the included somatic variant caller tools.

## Supporting Information

S1 Fig A pseudo measure of false positive calls returned by the somatic variant callers. For each caller, in exome sequencing and deep sequencing data in upper and lower panels, respectively, the diagrams depict whether mutations are called in only a single tumor sample or are reported recurrently from more than one tumor sample. Recurrent variant calls among the different tumor samples most likely represents false positive calls rather than a biological phenomenon and major differences are seen among the different somatic variant callers in this parameter.
(TIF)

S1 File Short description of the evaluated somatic variant callers. (DOCX)

S1 Table Mean coverage in exome sequencing and targeted deep sequencing. (DOCX)
