# Supplementary material for: Evaluation of Nine Somatic Variant Callers for Detection of Somatic Mutations in Exome and Targeted Deep Sequencing Data
Source: PLoS One. 2016 Mar 22;11(3):e0151664. doi: 10.1371/journal.pone.0151664 (PMC4803342; doi:10.1371/journal.pone.0151664)
Supplement: S1 File — (DOCX) [file pone.0151664.s002.docx]

**S1 File. Short description of the evaluated somatic variant callers.**

**Mutect**  is a somatic variant caller developed by Broad Institute (1) and is based on the Bayesian approach using a hidden Markov Model. The Mutect algorithm is divided into four steps: Removal of low-quality sequence data, variant detection in the tumor sample using a Bayesian classifier, filtering to remove false positives resulting from correlated sequencing artifacts that are not captured by the error-model and finally designation of the variants as somatic or germline by a second Bayesian classifier (1).

**Somatic Sniper,** developed by researchers at The Genome Institute at Washington University, is like Mutect based on a Bayesian posterior possibility (2). Somatic Sniper reports a somatic score (SSC), a Phred-scaled probability between 0 and 255, that the tumor and normal genotypes are different, where 0 means that there is no probability that they are different. We chose a quite strict cut-off value SSC > 40.

**Strelka** is a somatic variant caller developed by researchers at Illumina (3). Strelka produces separate VCF files containing single nucleotide variants (SNVs) and indels. The Strelka algorithm is a novel Bayesian approach wherein the tumor and normal allele frequencies are treated as continuous values (3). The first step in the software workflow is a search for candidate indels. Then reads are realigned and following realignment, the algorithm uses the read alignment information from both normal and tumor samples to produce somatic variant probabilities. Strelka uses allele frequencies rather than diploid genotypes, as it acknowledges that the normal tissue is a mixture of germline variation with noise and the tumor sample is a mixture of cancer cells with somatic mutations and normal tissue. As a final step in the Strelka workflow post-call filters are applied to the raw SNV and indel calls in order to minimize the number of false positives.

**Seurat** is a somatic variant caller developed by researchers from Phoenix, USA (4). Seurat is also based on a Bayesian algorithm and calculates the joint posterior probability that a variant exists in the tumor sample and not in the normal sample. The resulting VCF file contains both SNVs and indels.

**EBCall** (Empirical Bayesian mutation Calling) is a somatic variant caller developed by researchers from Tokyo (5). This variant caller is based on Bayesian theory and in addition uses sequencing data from multiple non-paired normal samples as prior knowledge of the distribution of sequencing errors. We utilized 36 exome sequencing BAM-files originating from normal tissue (blood), run under similar conditions as our study samples. In order to reduce the computational burden, only positions covered by more than seven reads from each strand in both normal and tumor sample are included and the variant shall be supported by at least three reads (5). The allele frequencies from the paired normal-tumor samples are then compared to the inferred sequencing error distribution and the algorithm uses this information to decide whether a somatic variant should be called. The EB Call VCF file contains both SNVs and indels.

**Virmid** is a somatic variant caller developed by a conjunction of researchers from California, USA and researchers from Korea (6). The first step in the Virmid algorithm is to estimate α, the level of impurity, i.e. the admixture of stromal cells in the cancer sample. A maximum likelihood estimation method is used. Next, the most probable genotype is estimated in the somatic variant caller step, using a Bayesian algorithm. The output VCF file from Virmid contains only SNVs.

**VarScan 2** is a somatic variant caller based on both a heuristic and statistical algorithm and is, like Somatic Sniper, developed at The Genome Institute at Washington University (7). Varscan 2 produces separate VCF files containing SNVs and indels. The Varscan 2 algorithm reads BAM files from tumor and normal samples simultaneously and performs heuristic pairwise comparisons of base calls and normalized sequence depths at each position. Variants are classified into germline, somatic, LOH and unknown which in the VCF files is stated as SS, variant status, as in Somatic Sniper. Variants are, by default, called homozygous if supported by 75 % or more of all reads at a position, otherwise they are called heterozygous (7). If the genotypes of the tumor and normal samples do not match at any given position, their read counts are evaluated by one-tailed Fishers exact test. The number of reference-supporting reads (outcome 1) and variant-supporting reads (outcome 2) observed in tumor (category 1) is compared to the same observed in normal (category 2). If the resulting p–value meets a significance threshold (user-defined) the variant is called somatic (if the normal matches the reference) or LOH (if the normal is heterozygous) (7). If the difference does not meet the significance threshold the variant is categorized as germline. The Fisher’s p-value is included in the VCF file as SPV (Somatic P-value for somatic/LOH events) and SSC (phred-scaled somatic score). The Fisher’s exact test is done for each variant and the p-values are not subsequently corrected for multiple testing. We utilized a cut-off value of Fisher’s p-value < 0.05.

**Shimmer** employs a statistical model quite similar to that of Varscan 2, but in addition to this it performs a correction for multiple testing. The Shimmer algorithm examines the base counts for each possible allele at every genomic position covered by sequence data in both the normal and tumor sample (8). If the total number of reads having a non-reference allele in tumor and normal exceeds a minimum threshold, a Fisher’s exact test is performed to test the null-hypothesis that there is no somatic mutation at this position. If the resulting p-value is below the cut-off the null-hypothesis can be rejected, stating that there is a somatic mutation at this position. As many p-values are created, subsequently, a correction for multiple testing is performed using the Benjamini and Hochberg algorithm (9). Hence, Shimmer VCF files only contain variant calls with a False Discovery Rate below a certain threshold.

**Indelocator** is a somatic variant caller tool developed by the Cancer Group at Broad Institute, like Mutect, but this tool is solely for detection of indels in cancer samples (not published).

**References**

1. Cibulskis K, Lawrence MS, Carter SL, Sivachenko A, Jaffe D, Sougnez C, et al. Sensitive detection of somatic point mutations in impure and heterogeneous cancer samples. NatBiotechnol. Marts 2013;31(1546-1696):213–9.

2. Larson DE, Harris CC, Chen K, Koboldt DC, Abbott TE, Dooling DJ, et al. SomaticSniper: identification of somatic point mutations in whole genome sequencing data. Bioinformatics. 1. Februar 2012;28(1367-4811):311–7.

3. Saunders CT, Wong WSW, Swamy S, Becq J, Murray LJ, Cheetham RK. Strelka: accurate somatic small-variant calling from sequenced tumor–normal sample pairs. Bioinformatics. 15. Juli 2012;28(14):1811–7.

4. Christoforides A, Carpten JD, Weiss GJ, Demeure MJ, Von Hoff DD, Craig DW. Identification of somatic mutations in cancer through Bayesian-based analysis of sequenced genome pairs. BMC Genomics. 4. Maj 2013;14:302.

5. Shiraishi Y, Sato Y, Chiba K, Okuno Y, Nagata Y, Yoshida K, et al. An empirical Bayesian framework for somatic mutation detection from cancer genome sequencing data. Nucleic Acids Res. April 2013;41(1362-4962):e89.

6. Kim S, Jeong K, Bhutani K, Lee JH, Patel A, Scott E, et al. Virmid: accurate detection of somatic mutations with sample impurity inference. Genome Biol. 2013;14(8):R90.

7. Koboldt DC, Zhang Q, Larson DE, Shen D, McLellan MD, Lin L, et al. VarScan 2: Somatic mutation and copy number alteration discovery in cancer by exome sequencing. Genome Res. 3. Januar 2012;22(3):568–76.

8. Hansen NF, Gartner JJ, Mei L, Samuels Y, Mullikin JC. Shimmer: detection of genetic alterations in tumors using next-generation sequence data. Bioinformatics. 15. Juni 2013;29(12):1498–503.

9. Benjamini and Hochberg Y. Controlling the false discovery rate: a practical and powerfull approach to multiple testing. JR Soc Stat B 57 289-300. 1995.
