# Supplementary material for: Evaluation of Nine Somatic Variant Callers for Detection of Somatic Mutations in Exome and Targeted Deep Sequencing Data
Source: PLoS One. 2016 Mar 22;11(3):e0151664. doi: 10.1371/journal.pone.0151664 (PMC4803342; doi:10.1371/journal.pone.0151664)
Supplement: S1 Table — (DOCX) [file pone.0151664.s003.docx]

| Sample | Mean coverage  Exome sequencing | Mean coverage  Targeted deep sequencing |
| --- | --- | --- |
| PTID 1 normal tissue | **76 x** | **320 x** |
| PTID 1 primary tumor | **89 x** | **415 x** |
| PTID 2 normal tissue | **148 x** | **330 x** |
| PTID 2 DCIS 1 | **98 x** | **484 x** |
| PTID 3 normal tissue | **64 x** | **233 x** |
| PTID 3 primary tumor | **72 x** | **457 x** |
| PTID 4 normal tissue | **49 x** | **148 x** |
| PTID 4 primary tumor | **73 x** | **453 x** |
| PTID 5 normal tissue | **45 x** | **338 x** |
| PTID 5 primary tumor | **83 x** | **447 x** |

**S1 Table. Mean coverage in exome sequencing and targeted deep sequencing.**
